# When the Fight against Fungi Goes Wrong

**DOI:** 10.1371/journal.ppat.1005400

**Published:** 2016-02-04

**Authors:** Xiaowen Wang, Frank L. van de Veerdonk

**Affiliations:** 1 Department of Dermatology, Peking University First Hospital, Beijing, China; 2 Department of Internal Medicine, Radboud Center for Infectious Diseases (RCI), Radboudumc, Nijmegen, The Netherlands; McGill University, CANADA

## Introduction

Fungi are associated with a wide spectrum of diseases in humans, with increasing morbidity and mortality [1]. Therefore, it is of great importance to elucidate the genetic and immunological mechanisms underlying the susceptibility to fungal infections. Recent studies of primary immunodeficiencies (PIDs)—a group of hereditary immune disorders with increased susceptibility to infection—have led to significant breakthroughs in our understanding of cellular and molecular mechanisms that predispose to both invasive and mucocutaneous fungal infections. This knowledge will pave the way for developing novel immunotherapeutic strategies in the near future.

## Identifying the Cause of PIDs

Chronic mucocutaneous candidiasis (CMC) is characterized by recurrent or persistent *Candida* infections of the skin, nails, and mucosal membranes. CMC may generally present as a distinct clinical entity, in which CMC is the only or main manifestation (called isolated CMC or CMC disease). CMC could also present as one of the manifestations in a syndrome (called syndromic CMC). Moreover, CMC can be observed in patients with acquired or inherited immunodeficiencies in addition to other infections [2]. By combining functional assays and focused genetic screening, causative mutations in two PIDs associated with CMC were discovered, namely in autosomal dominant hyper IgE syndrome (AD-HIES) and autosomal dominant CMC (AD-CMC). AD-HIES is a PID characterized by CMC, elevated serum IgE, eosinophilia, eczema, skeletal abnormalities, and recurrent staphylococcal infections; it was first described as Job’s syndrome in 1966 [3]. Minegishi et al. hypothesized that since a Tyk2 mutation had been found in a patient with a similar clinical syndrome to hyper IgE syndrome (HIES), cytokine signaling pathways that are dependent on Tyk2 might be deficient in AD-HIES [4]. They identified defective interleukin (IL)-10 signaling and IL-6 signaling and explored mutations in signal transducer and activator of transcription 3 (STAT3), which is a critical component in these signaling pathways. Eight out of 15 patients were found to have a loss of function (LOF) mutation in STAT3, which is now known to be responsible for the cause of disease in 60%–70% of patients with AD-HIES [4,5]. STAT3, activated by IL-6, IL-21, and IL-23, is essential for T helper (Th) 17 cell development because it provides signal transduction that induces transcription of RORγt, which in turn is crucial for the induction of Th17 cells. Indeed, patients harboring the dominant-negative STAT3 mutation have impaired Th17 cell responses to fungal infections [4,5]. This was the first observation linking deficient Th17 responses to CMC in patients.

In a similar way, mutations in STAT1 were found to underlie AD-CMC. The defective Th responses observed in AD-CMC were further explored by investigating cytokine-signaling pathways that drive Th1 and Th17 responses. IL-12 signaling, which is important for the induction of Th1 responses, and IL-23 signaling, which drives Th17 responses, were both found to be defective [6]. Molecules shared by these two pathways, such as receptor units and downstream molecules including STATs, were sequenced in five families with AD-CMC by next generation sequencing. All patients carried a mutation in the coiled-coil domain of STAT1. These mutations were found to be gain-of-function (GOF), which leads to hyperphosphorylation of STAT1 and accumulation of phosphorylated STAT1 in the nucleus [6,7]. Although the exact mechanism that is responsible for deficient Th17 responses remains to be elucidated, it is hypothesized that this is the result of an increased function of cytokines that dampen the Th17 response or due to less availability of STAT1 molecules to form heterodimers with other STAT molecules [7]. STAT1 GOF mutation is the most common hereditary cause of isolated CMC [6–8], and these mutations are associated with a spectrum of fungal infections, such as cutaneous fusariosis, disseminated coccidioidomycosis and histoplasmosis, *Penicillium marneffei* infections, and disseminated mucormycosis [9–12], underscoring the importance of STAT1-dependent responses in antifungal host defense.

## The Importance of the IL-17 Pathway in Antifungal Host Defense

Defective Th17 responses associated with the above two PIDs provided evidence that mucosal antifungal host responses are critically dependent on Th17 responses. IL-17A and IL-17F are key members of the IL-17 family and are produced predominantly by Th17 cells, although neutrophils and innate lymphoid cells can also produce these cytokines. IL-17 cytokines can recruit neutrophils and activate epithelial cells to produce defensins. To elicit their functions, IL-17A and IL-17F bind to IL-17RA/RC heterodimer complex, which subsequently triggers Act1-dependent NF-κB activation [13]. Mutations in *IL-17F* and its signaling pathway cause CMC, providing proof that the predominant pathway in mucosal antifungal host defense is the IL-17 pathway. One study reported autosomal recessive (AR) deficiency in *IL-17RA* and AD deficiency of *IL-17F* in CMC patients. *IL-17RA* deficiency was shown to be complete, abolishing cellular responses to IL-17A and IL-17F signaling. On the contrary, *IL-17F* deficiency was partial, with mutant IL-17F displaying impaired activity [14]. Recently, three patients from unrelated kindreds of CMC were reported to have AR *IL-17RC* deficiency. The patients were homozygous for different nonsense alleles that abolish the expression of IL-17RC, which prevented IL-17A and IL-17F signaling [15]. Moreover, IL-17R signaling is dependent on Act1 and a family with missense mutations in Act1, leading to defective IL-17 signaling and CMC, which again highlights the importance of the IL-17 pathway [16].

The syndrome of autoimmune polyendocrinopathy-candidiasis-ectodermal dystrophy (APECED), or autoimmune polyendocrine syndrome 1 (APS-1), is a rare AR PID characterized by CMC that is often its earliest manifestation, in addition to multiple autoimmune endocrinopathies, hypoparathyroidism, and adrenal insufficiency. The genetic cause of APECED are mutations in the autoimmune regulator (AIRE) gene [17]. Loss of function of AIRE in patients leads to impaired central T cell tolerance, with the generation of neutralizing autoantibodies against IL-17A, IL-17F, and/or IL-22, which might account for CMC [18,19]. These immunodeficiencies collectively point to a fundamental role for IL-17 signaling in the protection against mucosal *Candida* infection in humans.

## Essential Host Mechanisms for Preventing Invasive Fungal Disease

In addition to well-known risk factors for invasive fungal infection, such as neutropenia and corticosteroid therapy, PIDs have provided more insight into other mechanisms that protect us against invasive fungal disease. The first PID that directly provided a crucial mechanistic insight was chronic granulomatous disease (CGD). CGD is a disease with the highest incidence of invasive *Aspergillus* infection, and is even associated with invasive infections caused by nonpathogenic fungi, such as *A*. *nidulans* [20]. CGD is caused by mutations in one of the subunits of the phagocyte NADPH oxidase complex, which is composed of flavocytochrome b in plasma membrane (subunit gp91^phox^ and p22^phox^) and other cytosolic proteins (subunit p47^phox^, p67^phox^, p40^phox^, and small G-protein Rac1/2) [21,22]. Loss or functional inactivation of the NADPH oxidase complex results in the inability to produce reactive oxygen species (ROS), which are crucial for phagocytic killing of pathogens. Despite the ROS deficiency, CGD patients exhibit a hyperinflammatory state that leads to the formation of granulomas and inflammatory colitis. It was recently discovered that NADPH-dependent ROS deficiency results in autophagic dysfunction in monocyte/macrophages. As a result, autophagy-dependent IL-1β inhibition is impaired, with increased activation of IL-1β inflammasome, which may contribute to IL-1-mediated inflammation in CGD patients [23]. The defect of autophagy observed in monocytes from CGD patients is a form of noncanonical autophagy, called LC3-associated phagocytosis (LAP), which is important for killing *Aspergillus* [24,25].

The more recently discovered caspase recruitment domain-containing protein 9 (*CARD9*) deficiency provided novel insights in the role of this protein in invasive fungal infection and CMC. CARD9 is a key adaptor molecule expressed in myeloid cells downstream of the pattern recognition receptors (PRRs), Dectin-1, Dectin-2, and Mincle, which all recognize fungal cell wall components and subsequently activate spleen tyrosine kinase (SYK), which then engages CARD9 [26]. AR deficiencies were discovered to cause both CMC and *Candida* meningoencephalitis [27–30]. In addition, patients with idiopathic deep dermatophytosis, subcutaneous pheohyphomycosis, and invasive *Exophiala* infections were also reported to have AR *CARD9* deficiency [31–33]. CARD9 deficiency not only results in an insufficient *Candida*-induced Th17 response [27] but neutrophils isolated from these patients also display a selective *Candida albicans* killing defect [28], which explains the susceptibility to both mucocutaneous and invasive antifungal host defense. These two PIDs underscore two crucial mechanisms to control severe invasive fungal infection, namely the NADPH oxidase complex and CARD9-dependent signaling.

## Designing Novel Targeted Therapeutic Strategies

**Fig 1:**
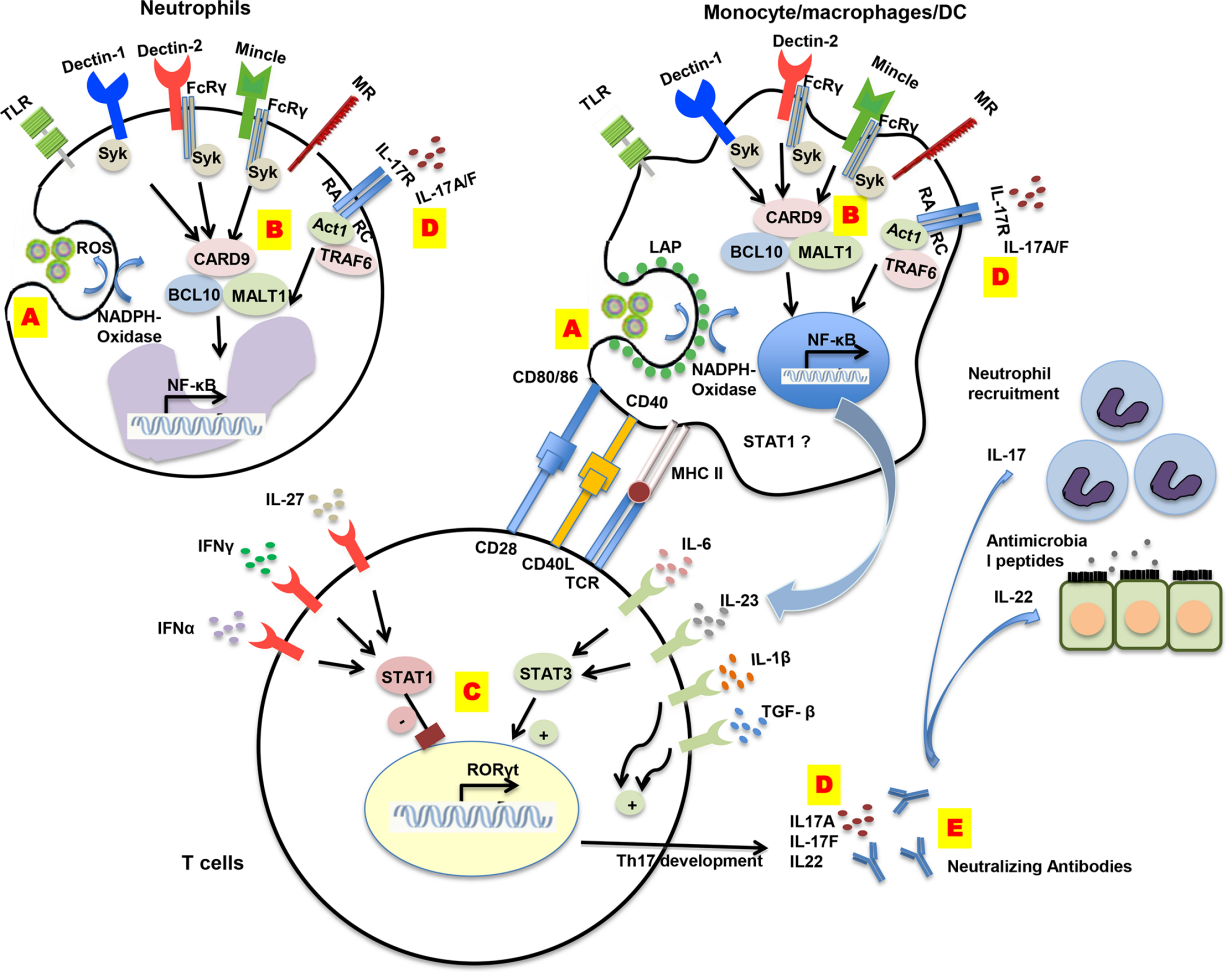
Schematic overview of crucial mechanisms in antifungal host defense. **(A) NADPH oxidase:** This protein complex is responsible for reactive oxygen species (ROS) production as well as LC3-associated phagocytosis (LAP), which both play a role in fungal clearance. **(B) CARD9-dependent PRR pathways:** After pattern recognition, downstream signaling passes through adaptor molecule CARD9, forming a complex with BCL-10 and MALT1, which drives NF-κB responses. Proinflammatory cytokines, such as IL-1β, IL-6, IL-23, and TGF-β, are secreted. **(C) STAT1 and STAT3:** Proinflammatory cytokines signal through STAT3, which induces transcription of RORγt, leading to differentiation of naive T cells towards Th17 cell lineage. STAT1 gain-of-function may shift the cellular response from STAT3-mediated Th17 cell-activating cytokines toward hyper-responses of Th17 inhibiting cytokines, such as IL-27, interferon (IFN)-γ, and IFN-α. **(D) IL-17 and IL-17 signaling:** As key adaptive cytokines in host defense against fungi, IL-17A and IL-17F signal through the IL-17RA/RC heterodimer complex, forming IL-17R-Act1-TRAF6 complex to trigger NF-κB activation. Therefore, patients with IL-17F, IL-17RA, IL-17RC, or Act1 deficiencies have either impaired IL-17 function or impaired IL-17 signaling responses. **(E) Autoantibodies against cytokines:** Patients with AIRE deficiency develop high levels of neutralizing autoantibodies against IL-17A, IL-17F, and/or IL-22, which directly antagonize IL-17 and IL-22 responses. DC, dendritic cell; TLR, toll-like receptor; MR, mannose receptor; FcγR, Fcγ receptor; CARD9, caspase recruitment domain-containing protein 9; Syk, spleen tyrosine kinase; BCL-10, B cell lymphoma/leukemia 10; MALT1, mucosa-associated lymphoid tissue lymphoma translocation protein 1; NF-κB, nuclear factor-κB; STAT, signal transducer and activator of transcription; MHC II, major histocompatibility complex class II; TCR, T cell receptor; IL, interleukin; IFN, interferon; RORγt, retinoic acid-related orphan receptor gamma t; Th17 cell, T helper 17 cell; RA, receptor A; TRAF6, tumor necrosis factor (TNF) receptor-associated factor 6.

The main mechanisms in fungal infections discovered via PIDs are summarized in Table 1 and Fig 1. With this knowledge comes the challenge of translating these findings into therapeutic strategies that can improve morbidity and mortality in PID patients with fungal infection. Decreasing STAT1 activity in patients with a *STAT1* GOF function mutation would be a rationale for targeted therapy in these patients. Recent findings suggest that direct STAT1 inhibition with fludarabine can reverse the reduced STAT3-dependent gene transcription that is observed in CMC cells in vitro; however, whether this treatment would be beneficial in CMC is currently unknown [34]. What supports this strategy is that inhibition of cytokine-induced STAT1 activity by a JAK1/JAK2 inhibitor was beneficial in a patient with AD-CMC [35].

The autoantibodies against IL-17 and IL-22 observed in APECED might be targeted by B cell or plasma cell-depletion strategies, since this has also been observed to be helpful in patients suffering from nonmycobacterial disease due to autoantibodies against IFNγ. Interestingly, neutralizing autoantibodies against IFNγ and granulocyte macrophage colony-stimulating factor (GM-CSF) have also been associated with invasive penicilliosis [36] and cryptococcal meningitis [37], respectively, providing a rationale to explore B cell depleting therapies in these circumstances. The recognition of autoantibodies against cytokines causing fungal disease not only opens up new treatment strategies in these rare diseases, but also demonstrates the importance of these cytokines in antifungal host defense. Indeed, immunomodulatory therapies with these recombinant cytokines, in addition to antifungal treatment in patients with PIDs, have been used with success. Patients with CGD can have benefits from recombinant IFNγ therapy: A patient with a *Candida* meningoencephalitis due to CARD9 deficiency was successfully treated with GM-CSF, and one patient with *STAT1* GOF mutation was successfully treated with GM-CSF initially and later G-CSF [29,38]. Also, blocking cytokines in CGD has been explored, which has led to the observation that IL-1Ra is not only able to dampen IL-1β-mediated inflammation in CGD, but it also restores defective LAP-mediated *Aspergillus* clearing, suggesting that anakinra might serve as a promising adjunctive treatment option in CGD patients during fungal infection [23].

**Table 1:** Summary of genes involved in PIDs with fungal infections.

Gene	Mode of inheritance	Disease	Associated fungal pathogens	Immunological phenotype	Refs.
***STAT3***	AD	AD-HIES	*Candida*	Impaired Th17 differentiation	[4,5]
***STAT1***	AD	AD-CMC, cutaneous fusariosis, disseminated coccidioidomycosis and histoplasmosis, *Penicillium marneffei* infections, and disseminated mucormycosis	*Candida*, *Fusarium*, *Coccidioides*, *Histoplasma*, *Penicillium marneffei*, *Apophysomyces*	Hyperphosphorylation of STAT1, deficient Th17 responses	[6–12]
***IL17F***	AD	CMC	*Candida*	Defective IL-17F bioactivity	[14]
***IL17RA/C***	AR	CMC	*Candida*	Lack of cellular responses to IL-17A and IL-17F	[14,15]
***ACT1***	AR	CMC	*Candida*	Impaired IL-17 signaling	[16]
***AIRE***	AR	APECED	*Candida*	Autoantibodies against IL-17 and IL-22	[17]
***CYBB***	X-linked	CGD	*Candida*, *Aspergillus*	NADPH oxidase complex deficiency	[21]
***NCF1*, *NCF2*, *NCF4*, *CYBA***	AR	CGD	*Candida*, *Aspergillus*	NADPH oxidase complex deficiency	[21]
***CARD9***	AR	CMC, *Candida* Meningoencephalitis, deep dermatophytosis, subcutaneous pheohyphomycosis, and invasive *Exophiala* infections	*Candida*, *Trichophyton*, *Phialophora*, *Exophiala*	Reduced TNF-α production and circulating IL-17-producing T cells, killing defect of neutrophils	[27–33]
***DOCK8***	AR	AR-HIES	*Candida*	Impaired Th17 differentiation	[41]
***RORC***	AR	Candidiasis and mycobacteriosis	*Candida*	Absence of IL-17A/F-producing T cells	[42]
***IL-12RB1***	AR	CMC, Mycobacterial and *Salmonella* infections	*Candida*	Loss of function of IL-12 and IL-23 receptor, diminished IFN-γ and IL-17	[43]
***TYK2***	AR	HIES, mycobacterial and viral infections	*Candida*	Reduced Th1 and type I IFN responses	[44]

## System Biology Approach and Future Directions

By performing transcriptomics on human cells exposed to various stimuli, it was discovered that *Candida* induces a strong type I IFN signature, which is typically associated with antiviral host responses [39]. The importance of this finding was reflected by the association of polymorphisms in genes regulating the IFN pathway with susceptibility to candidemia (such as *MDA5*, which is a viral PRR [40]). This approach led to exploring novel pathways that would otherwise not be so logical to investigate in patients with fungal infection. This system biology approach might also help to understand several observations made in patients with PIDs in recent years. There is the observation that AR-HIES can be caused by mutations in the dedicator of cytokinesis 8 (*DOCK8*) gene, which results in both increased susceptibility to recurrent viral infections and CMC [41]. Moreover, it is striking that genes that are associated with susceptibility to nonmycobacterial disease are also associated with fungal infection. Recently, homozygous loss-of-function mutations in *RORC*, which encodes RORγ that is important for the induction of Th17 cells, have been described to cause increased susceptibility to mycobacterial infection and *Candida* infections [42]. Mutations of *IL12RB1* and tyrosine kinase 2 (*Tyk2*) genes, impairing the IL-12/IFNγ axis, also predisposes to both CMC and increased susceptibility to mycobacterial disease [43,44]. In light of these observations, *STAT1* mutations are the most intriguing. A loss of function of STAT1 leads to increased susceptibility to mycobacterial disease [45], whereas a GOF mutation leads to CMC and invasive endemic fungal infection. These observations reflect how important a well-balanced IFN pathway and STAT1 activity is to preventing viral, mycobacterial, and fungal infections, and they open up a new field to explore in patients with fungal infection.

## Conclusion

In the past decade, studies of PIDs have hugely promoted our understanding of the immunological pathways involved in human antifungal immunity. This knowledge helped to elucidate mechanisms that play a crucial role in antifungal host defense and offered unique opportunities to link clinical phenotypes to immunological function. We have learned from the described immunodeficiencies that the IL-17 pathway is fundamental for mucosal antifungal host defense, while neutrophil function and IFNγ and GM-CSF are essential for preventing invasive fungal infection. Of course, our present knowledge is still limited, and there are a large number of fungal infections for which the environmental and genetic background has yet to be deciphered. We believe that a joint effort from the field of immunology, genetics, microbiology, and systems biology will provide new insight into host immune response against fungi, which will facilitate the development of personalized immunotherapeutic strategies in fungal infection.
